# Dose-volume analysis of predictors for acute anal toxicity after radiotherapy in prostate cancer patients

**DOI:** 10.1186/s13014-019-1374-1

**Published:** 2019-10-10

**Authors:** Xingsi Peng, Sha Zhou, Shiliang Liu, Jibin Li, Sijuan Huang, Xiaobo Jiang, Maosheng Lin, Shaomin Huang, Chengguang Lin, Chaonan Qian, Mengzhong Liu, Liru He

**Affiliations:** 1 Department of Radiation Oncology, Sun Yat-Sen University Cancer Center, State Key Laboratory of Oncology in South China, Collaborative Innovation Center for Cancer Medicine, Guangzhou, China; 2 Department of Nasopharyngeal Carcinoma, Sun Yat-sen University Cancer Center; State Key Laboratory of Oncology in South China; Collaborative Innovation Center of Cancer Medicine, Guangzhou, Guangdong 510060 People’s Republic of China; 3 Department of Clinical Research, Sun Yat-Sen University Cancer Center, State Key Laboratory of Oncology in South China, Collaborative Innovation Center for Cancer Medicine, Guangzhou, China

**Keywords:** Prostate cancer, Radiotherapy, Hemorrhoid, Acute anal toxicity

## Abstract

**Background:**

This study aimed to evaluate the clinical and dosimetric factors predictive of acute anal toxicity (AAT) after radiotherapy in prostate cancer (PCa) patients with or without hemorrhoids.

**Methods:**

We analyzed data from 347 PCa patients (248 cases treated from July 2013 to November 2017 for training cohort and 99 cases treated in 2018 for validation cohort) treated with pelvic radiotherapy at a single institution. Anal canal dose–volume histogram was used to determine the prescribed dose. Univariate and multivariate analyses were used to evaluate the risk of AAT as a function of clinical and dosimetric factors.

**Results:**

Totally, 39.5% (98/248) and 31.3% (31/99) of the PCa patients developed AAT in training and validation cohorts, respectively. The incidence of AAT was much higher in patients with hemorrhoids than in those without hemorrhoids in both training and validation cohorts. Hemorrhoids and volume received more than 20 Gy (V20) were valuated as independent factors for predicting AAT in training cohort. Similar results were also observed in our validation cohort. The combination of hemorrhoids and high anal canal V20 (> 74.93% as determined by ROC curves) showed the highest specificity and positive predictive values for predicting AAT in both training and validation cohorts.

**Conclusions:**

AAT occurs commonly in PCa patients with hemorrhoids during and after pelvic radiotherapy. Hemorrhoids and anal canal V20 are independent predictors of AAT. These factors should be carefully considered during treatment planning to minimize the incidence of AAT.

## Introduction

Prostate cancer (PCa) is the most frequently diagnosed cancer among men in America and Europe, and its incidence is rapidly increasing in Asian counties, including China [1–4]. With the increasing use of radiotherapy (RT) in the curative management of this disease over the last few decades, the potential radiotoxicity has drawn increased attention.

With respect to gastrointestinal toxicity, a number of reports have described rectal [5–7] or anorectal toxicity [8–10], which manifests in symptoms such as acute diarrhea, fecal incontinence, chronic proctitis, and rectal bleeding. Symptoms of acute anal toxicity (AAT), especially anal pain or bleeding, tend to be ignored because of their relatively lower severities. However, anal pain and bleeding are quite common symptoms during pelvic radiotherapy for Asian patients. Two studies from Korea indicated that hemorrhoids are an important risk factor for AAT in patients undergoing pelvic radiotherapy [11, 12]. However, both studies only enrolled small numbers of patients (31 and 33 patients, respectively) and included cases with different cancers and different radiation techniques, which made these data inadequate for reliable dosimetric analysis.

The prevalence of hemorrhoids is reported to be up to 90% in China [13], which is much higher than that in Western Europe and the United States [14, 15]. Chinese patients with PCa show a much higher risk of AAT after radiotherapy. However, we still have no clear idea about how to help the hemorrhoids patients to reduce the risk of AAT. And the dosimetric recommendations of anal canal for preventing AAT have long been lacking. The purpose of our study was to determine the risk factors, in particular the dosimetric factors, for AAT in Chinese PCa patients who were treated with radiotherapy at our institution.

## Methods

### Patient selection

The institutional review board approved this retrospective study and waived the need to obtain informed consent. We retrospectively reviewed 248 PCa patients treated at the Radiation Oncology Department of our institution between July 2013 and November 2017. As a validation, 99 PCa patients treated with pelvic radiotherapy in 2018 at the same institution were also studied. The following criteria were used to select the patients for our study: pathologically confirmed diagnosis of prostatic adenocarcinoma; definitive radiotherapy to the primary tumor or postoperative radiotherapy to the tumor bed; the availability of anal dose–volume histograms (DVHs) in our institutional archives; and the availability of weekly patient records during the treatment and post-radiotherapy clinical assessments of patients’ AAT. Exclusion criteria were previous pelvic radiotherapy, secondary malignancy, and unknown critical clinical information.

### Simulation and contouring

Simulation CT was performed with 3-mm slices in the prone position on a belly board. All the patients had made dietary preparation at least 1 week prior to the day of the CT scan. And cone beam CT or portal imaging was performed in general for the patients. The CT image was transferred to the workstation (Monaco V5.11, Elekta AB, Stockholm, Sweden), and the target volumes and critical organs were contoured. Clinical target volumes were contoured according to the recommendations of the Radiation Therapy Oncology Group (RTOG). Clinical target volumes were expanded by 5–7 mm (3–5 mm posterior) to produce planning target volumes (PTVs). The location of the anal verge was defined on the basis of the last caudal image of the external sphincter muscle in CT images. The anal canal volume was defined as the volume from the anal verge to 3 cm superior to the anal verge in planning CT images.

### Treatment approaches

Patients with intermediate, high, or very high-risk PCa and patients underwent salvage radiotherapy postoperatively received androgen deprivation therapy before, during, and after radiotherapy. Patients with low-risk PCa or those treated postoperatively with an adjuvant intent received radiotherapy alone. Patients with low risk did not receive pelvic RT. Postoperative patients with regional lymph nodes metastasis received pelvic RT. The radiation doses for the pelvic were 45~50Gy/25F. The conventional fraction modality (1.8–2.0 Gy per fraction) was usually used to treat PCa patients postoperatively, whereas the hypofractioned modality (2.4–3.0 Gy per fraction) was only prescribed to PCa patients with definitive treatment intent. Before October 2014, patients received intensity-modulated radiotherapy (IMRT) or volumetric-modulated arc therapy (VMAT), and after October 2014, all the patients were treated by image-guided IMRT or VMAT.

### Clinical evaluation and follow-up

During radiotherapy, symptoms were closely monitored on a weekly basis and even more frequently if required for clinical evaluation and disease management. After completing radiotherapy, all patients were followed up every 3 months for the first 2 years and every 6–12 months thereafter. The median follow-up time is 36 months. And all patients were followed up more than 3 months. As we observed in clinical assessments, the main symptoms of AAT were anal pain and anal bleeding. We graded AATs according to the National Cancer Institute-Common Terminology Criteria for Adverse Events version 4.03 (NCI-CTCv4.03) as follows: Grade 1: mild bleeding without intervention indicated and/or mild pain not interfering with function; Grade 2: symptomatic bleeding requiring medical intervention or minor cauterization, and/or moderate pain interfering with function, but not interfering with activities of daily living (ADLs); Grade 3: bleeding that required transfusion, interventional radiology, endoscopic, or operative intervention, and/or severe pain severely interfering with ADL; Grade 4: life-threatening bleeding consequences with major urgent intervention indicated and/or disabling pain; Grade 5: Death.

### Statistical analysis

Descriptive statistics (e.g., means, standard deviation, and percentages) were presented when appropriate. The differences in the clinical and DVH characteristics between the AAT and NAAT groups were compared using Mann–Whitney’s *U* test for quantitative variables and Fisher’s exact test for categorical variables. Multivariate logistic regression models were created to evaluate the risk of AAT as a function of clinical and dosimetric factors. The factors with *p* < 0.05 in univariate analyses were involved in subsequent multivariate analyses with forward stepwise analyses to assess the independent factors associated with the risk of AAT. Receiver operating characteristic (ROC) curve analyses were performed for DVH parameters to select the most relevant threshold to differentiate symptomatic AAT. The optimal threshold for each DVH parameter was defined as the point yielding the minimal value for (1 - sensitivity)^2^ + (1 - specificity)^2^, which was the point on the ROC curve closest to the upper left-hand corner (0, 1) [16]. A *P* value < 0.05 was considered statistically significant. All analyses were performed using SPSS (version 20.0, Chicago).

## Results

### Patient characteristics

The characteristics of the 248 patients from training cohort and 99 patients from validation cohort are summarized in Table 1. The median age of the two group patient cohorts was 67 years. One hundred and thirty-nine patients (56.0%) and 52 patients (52.5%) reported hemorrhoids before radiotherapy in training and validation cohorts, respectively. The majority (71.76%) of the patients received androgen deprivation therapy before, during, and after RT. The radiation dose per fraction ranged from 1.8 Gy and 3.0 Gy, and the total dose varied from 60 Gy to 81 Gy. The majority of the patients (303/347, 87.3%) received image-guided radiotherapy (IGRT), and the other patients received IMRT or VMAT. Portal imaging was performed before the initiation of the IMRT/VMAT course, while cone beam CT was performed before each treatment for patients treated with IGRT.

**Table 1:** Patient characteristics

	Training group (*N* = 248 cases)			Validation group (*N* = 99 cases)		
Factors	NAAT	AAT	*P*	NAAT	AAT	*P*
Age (%)						
46-68ys	72 (55.8)	57 (44.2)	0.117	44 (75.9)	14 (24.1)	0.067
68-88ys	78 (65.5)	41 (34.5)		24 (58.5)	17 (41.5)	
T stage (%)						
1~2	36 (56.3)	28 (43.7)	0.789	21 (67.7)	10 (32.3)	0.933
3~4	114 (62.0)	70 (38.0)		45 (69.2)	20 (30.8)	
N stage (%)						
0	92 (64.3)	51 (35.7)	0.148	40 (65.6)	21 (34.4)	0.375
1	58 (55.2)	47 (44.8)		26 (74.3)	9 (25.7)	
M stage (%)						
0	90 (60.4)	59 (39.6)	0.974	48 (69.6)	21 (30.4)	0.775
1	60 (60.6)	39 (39.4)		20 (66.7)	10 (33.3)	
Clinical stage (%)						
I~II	23 (54.8)	19 (45.2)	0.753	18 (66.7)	9 (33.3)	0.880
III~IV	127 (61.7)	79 (38.3)		50 (69.4)	22 (30.6)	
GS group (%)						
6~7	66 (64.7)	36 (35.3)	0.256	25 (80.6)	6 (19.4)	0.074
8~10	84 (57.5)	62 (42.5)		40 (62.5)	24 (37.5)	
TPSA before treatment (%)						
< 20 ng/mL	22 (59.5)	15 (40.5)	0.890	40 (62.5)	24 (37.5)	0.226
≥ 20 ng/mL	128 (60.7)	83 (39.3)		19 (76.0)	6 (24.0)	
TPSA before RT (%)·						
< 20 ng/mL	119 (60.1)	79 (39.9)	0.806	61 (67.0)	30 (33.0)	0.407
≥ 20 ng/mL	31 (62.0)	19 (28.0)		5 (83.3)	1 (16.7)	
RT type (%)						
IMRT/VMAT	23 (67.6)	11 (32.4)	0.358	5 (50.0)	5 (50.0)	0.179
IMRT/VMAT-IGRT	127 (59.3)	87 (40.7)		63 (70.8)	26 (29.2)	
RT modality (%)						
Conventional fraction	126 (62.1)	77 (37.9)	0.278	46 (66.7)	23 (33.3)	0.511
Hypo-fraction	24 (53.3)	21 (46.7)		22 (73.3)	8 (26.7)	
Surgery (%)						
No	94 (61.4)	59 (38.6)	0.697	31 (62.0)	19 (38.0)	0.483
Yes	56 (58.9)	39 (41.1)		33 (68.8)	15 (31.3)	
Hormonal treatment duration (%)						
< 2 years	23 (54.8)	19 (45.2)	0.405	17 (63.0)	10 (37.0)	0.730
≥ 2 years	127 (61.7)	79 (38.3)		48 (66.7)	24 (33.3)	
Hemorrhoid (%)						
NO	95 (87.2)	14 (12.8)	< 0.001	39 (83.0)	8 (17.0)	0.004
Yes	55 (39.6)	84 (60.4)		29 (55.8)	23 (44.2)	

Abbreviations: *GS* Gleason score; *TPSA* total prostate-specific antigen; *RT* radiation therapy

### Clinical characteristics and acute anal toxicity

Totally, 39.5% (98/248) and 31.3% (31/99) of the PCa patients developed AAT in training and validation cohorts, respectively. There were 89 (35.8%), 8 (3.2%) and 1(0.4%) patients developed grade 1, grade 2 and grade 3 AAT in training cohort. While 29 (29.3%), 2(2.0%) and 0(0.0%) of the patients reported grade 1, grade 2 and grade 3 AAT in validation cohort. No grade 4 or grade 5 AATs were observed in both cohorts. At the beginning of radiotherapy, the majority of the patients were asymptomatic, and only 15 patients reported mild anal pain and/or bleeding. All 15 symptomatic patients complained of anal symptom aggravation during the course of radiotherapy, including 3 patients (Grade 2 and 3 anal bleeding for 2 and 1 patients, respectively) who had to suspend radiotherapy for more than 2 weeks to receive medical intervention. The median time to the initiation of anal symptoms was 3 weeks (range, 0 to 5 weeks), with a corresponding median induction dose of about 37.5 Gy (EQD2:38.3Gy, α/β for anal canal 4).

In both cohorts, hemorrhoids were evaluated as the only clinical factor that showed a significant association with AAT (Table 1). The incidence of AAT was much higher in patients with hemorrhoids than in those without hemorrhoids in training cohort (60.4% vs. 12.8%, *P <* 0.001) and validation cohort (44.2% vs. 17.0%, *P =* 0.004). No significant differences were found between the no-acute anal toxicity (NAAT) groups and AAT groups with respect to the other clinical variables, such as age, T stage, N stage, M stage, clinical stage, GS group, TPSA before treatment or before RT, RT type, RT modality, Surgery and Hormonal treatment duration in both training and validation cohorts (Table 1).

### Dose–volume parameters and acute anal toxicity

In both training and validation cohorts, the mean prescribed dose of radiotherapy and the mean volume of the anal canal were not significantly different between the NAAT and AAT groups (Table 2). The maximum dose for the anal canal for the AAT group was significantly higher than those for the NAAT group in training cohort, while it was not significantly in validation cohort (*P* = 0.018 and *P* = 0.169, Table 2). In addition, in both cohorts, AAT patients showed significantly higher mean dose for anal canal, V10, V20, V30, V40, V50 and V60 values than NAAT patients (*P* < 0.05, Table 2).

**Table 2:** Univariate analysis of DVH parameters related to AAT

	Training group (*N* = 248 cases)			Validation group (*N* = 99 cases)		
Variables	NAAT (Mean ± SD)	AAT (Mean ± SD)	*P*	NAAT (Mean ± SD)	AAT (Mean ± SD)	*P*
Prescribed Dose (Gy)	67.04 ± 5.02	67.33 ± 5.77	0.672	69.12 ± 4.65	67.35 ± 3.96	0.073
Volume of anal canal (cm^3^)	9.43 ± 4.02	10.02 ± 3.60	0.232	9.33 ± 3.51	8.00 ± 3.17	0.080
Anal canal Dmax (Gy)	59.51 ± 12.95	63.22 ± 9.65	0.018	54.47 ± 13.83	58.40 ± 11.13	0.169
Anal canal Dmean (Gy)	26.04 ± 12.60	30.25 ± 9.71	0.010	22.51 ± 9.61	29.43 ± 11.52	0.005
V10 (%)	75.61 ± 22.45	84.48 ± 18.89	0.002	67.82 ± 22.35	85.52 ± 18.06	0.001
V20 (%)	58.41 ± 26.62	72.62 ± 25.59	< 0.001	49.71 ± 29.09	74.24 ± 23.84	< 0.001
V30 (%)	37.81 ± 24.32	52.08 ± 26.91	< 0.001	30.65 ± 27.74	49.85 ± 30.98	0.006
V40 (%)	19.31 ± 16.18	27.30 ± 20.44	0.001	13.75 ± 14.98	27.46 ± 25.60	0.009
V50 (%)	8.81 ± 9.09	13.40 ± 13.01	0.002	5.22 ± 6.40	12.83 ± 15.38	0.011
V60 (%)	3.16 ± 4.33	5.34 ± 7.28	0.006	1.66 ± 2.83	3.95 ± 5.59	0.038
V70 (%)	0.08 ± 0.31	0.44 ± 2.13	0.099	0.07 ± 0.24	0.05 ± 0.27	0.785

Abbreviations: *DVH* dose–volume histograms; *AAT* acute anal toxicity; *NAAT* no-acute anal toxicity; *Dmax* maximum dose; *Dmean* mean dose; *V10–V70* percentage of anal canal volume receiving more than 10–70 Gy

### Multivariate regression analysis of acute anal toxicity

Hemorrhoids and the dose–volume parameters factors that showed significance in univariate analysis were further analyzed in the multivariate regression model (Table 3). In both training and validation cohorts, only hemorrhoids (OR = 10.94 and 5.34, respectively, *P* < 0.001) and V20 (OR = 1.03 and 1.04, respectively, *P* < 0.001) were found to be independent predictors of AAT.

**Table 3:** Multivariate regression analysis related to AAT

Groups	Variables	OR (95% CI)	*P*
Training group (*N* = 248 cases)	V10 (%)	1.02 (1.01~1.03)	0.650
	V20 (%)	1.03 (1.01~1.04)	< 0.001
	V30 (%)	1.02 (1.01~1.03)	0.607
	V40 (%)	1.02 (1.01~1.04)	0.947
	V50 (%)	1.04 (1.01~1.07)	0.847
	V60 (%)	1.07 (1.02~1.13)	0.526
	Anal canal Dmax (Gy)	1.00 (0.96~1.04)	0.994
	Anal canal Dmean (Gy)	1.00 (0.96~1.05)	0.810
	Hemorrhoid (cases)	10.94 (5.47~21.89)	< 0.001
Validation group (*N* = 99 cases)	V10 (%)	1.04 (1.02~1.06)	0.478
	V20 (%)	1.04 (1.02~1.06)	< 0.001
	V30 (%)	1.02 (1.01~1.04)	0.461
	V40 (%)	1.03 (1.01~1.06)	0.676
	V50 (%)	1.06 (1.01~1.11)	0.438
	V60 (%)	1.12 (1.01~1.24)	0.559
	Anal canal Dmean (Gy)	1.06 (1.02~1.11)	0.712
	Hemorrhoid (cases)	5.34 (1.85~15.39)	< 0.001

Abbreviations: *AAT* acute anal toxicity; *OR* odds ratio; *CI* confidence interval; V10–V60 = percentage of anal canal volume receiving more than 10–60 Gy; Dmax = maximum dose; Dmean = minimum dose

### The anal canal V20 and the risk of acute anal toxicity

**Fig. 1:**
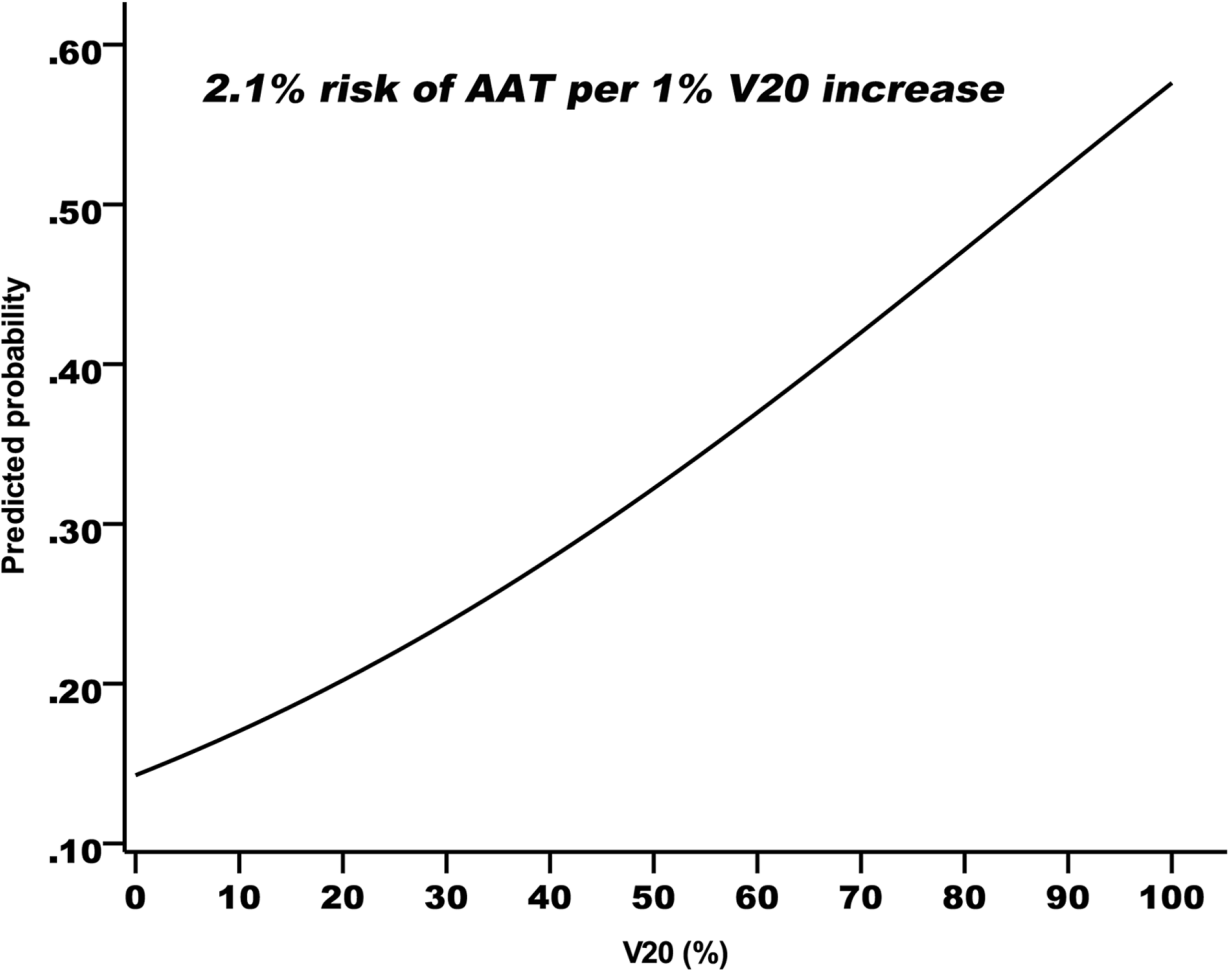
The predicted probability of radiation-related anal reaction as a function of the relative anal volume treated with 20 Gy radiation (V20) by using logistic regression model

To confirm the effect of the anal canal V20 on the risk of AAT, we grouped the patients from the training cohort into ten equal-sized bins in terms of patient number and plotted the incidence risk vs. the V20. The solid curve in Fig. 1 shows the fit of the logistic model to the data and the trend toward an increased risk of AAT with a higher anal canal V20. There is an average increase of 2.1% in AAT probability for each incremental 1% rise in the anal canal V20.

**Fig. 2:**
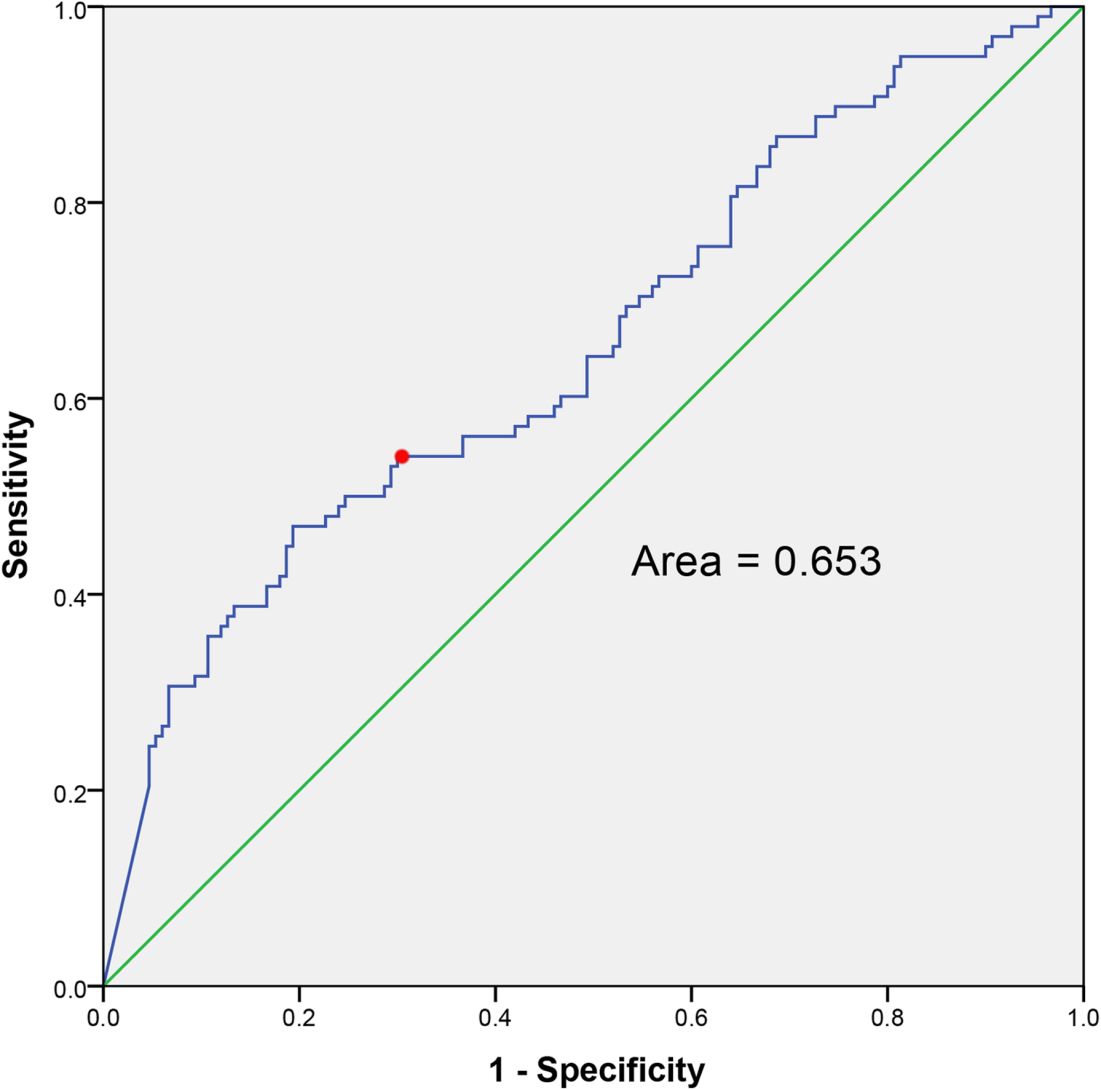
ROC curves of all patients and the associated area for V20 as a predictor for AAT. The optimal threshold value is 74.93% (plotted using red circle) and corresponds to a sensitivity of 0.541 and specificity of 0.700

ROC curve analysis was performed in the training cohort to select the most relevant dose–volume parameter to predict AAT. The optimal threshold of the anal canal V20, with an area under the ROC curve (AUC) of 0.653 (sensitivity: 0.541, specificity: 0.700, Fig. 2). The incidence of AAT was significantly higher in patients with an anal canal V20 more than 74.93% than in those with an anal canal V20 less than 74.93% (54.08% vs. 30.00%, *P* < 0.001).

### Predictive model analysis of acute anal toxicity

To identify the most optimal predictive model for AATs, we further analyzed the sensitivity, specificity, positive predictive value (+PV), negative predictive value (−PV), and AUC of hemorrhoids and anal canal V20 in both training cohort and validation cohort. As shown in Table 4, hemorrhoids showed the best sensitivity (85.71 and 74.19%) and –PV (87.16 and 82.98%) in predicting AAT, while the combination of hemorrhoids and anal canal V20 > 74.93% showed the highest specificity (91.33 and 94.12%) and + PV (77.97 and 75.00%) for predicting AAT in training and validation cohorts, respectively.

**Table 4:** Predictive value analysis of hemorrhoids and/or anal canal V20

Groups	Models	AAT (cases)	NAAT (cases)	Sensitivity (%)	Specificity(%)	+PV (%)	-PV (%)	AUC
Training group (*N* = 248 cases)	Model 1: Anal canal V20							
	High(V20 > 74.93%)	53	45	54.01	70.00	54.08	70.00	0.620
	Low(V20 ≤ 74.93%)	45	105					
	Model 2: Hemorrhoid							
	Yes	84	55	85.71	63.33	60.43	87.16	0.745
	No	14	95					
	Model 3:							
	High V20 with hemorrhoid	46	13	46.94	91.33	77.97	72.49	0.691
	Others	52	137					
	Model 4:							
	High V20 without hemorrhoid	7	32	7.14	78.67	17.95	56.46	0.429
	Others	91	118					
	Model 5:							
	Low V20 with hemorrhoid	38	42	38.78	72.00	47.50	68.35	0.554
	Others	50	108					
	Model 6:							
	Low V20 without hemorrhoid	7	63	7.14	58.00	10.00	48.88	0.326
	Others	91	87					
Validation group (*N* = 99 cases)	Model 1: Anal canal V20							
	High(V20 > 74.93%)	18	13	58.06	80.88	58.06	80.88	0.665
	Low(V20 ≤ 74.93%)	13	55					
	Model 2: Hemorrhoid							
	Yes	23	29	74.19	57.35	44.23	82.98	0.658
	No	8	39					
	Model 3:							
	High V20 with hemorrhoid	11	4	35.48	94.12	75.00	76.19	0.631
	Others	20	64					
	Model 4:							
	High V20 without hemorrhoid	7	9	22.58	86.76	43.75	71.08	0.534
	Others	24	59					
	Model 5:							
	Low V20 with hemorrhoid	14	23	45.16	66.18	37.84	72.58	0.529
	Others	17	45					
	Model 6:							
	Low V20 without hemorrhoid	2	29	6.45	57.35	6.45	57.35	0.306
	Others	29	39					

Abbreviations: *V20* percentage of anal canal volume receiving more than 20 Gy; *AAT* acute anal toxicity; *NAAT* no-acute anal toxicity; *+PV* positive predictive value; *−PV* negative predictive value; *AUC* area under the curve

## Discussion

One of the important findings in this study is that hemorrhoids are the most important risk factor for predicting AAT. After the beginning of pelvic irradiation, anal symptoms developed or worsened in 84 (60.4%) of 139 patients with hemorrhoids and only in 14 (12.8%) of 109 patients without hemorrhoids. Similar results were also observed in validation cohort, in which AAT developed in 44.2% of the patients with hemorrhoids, but only in 17.0% of the patients without hemorrhoids. Our results also confirm the high frequency of AAT (42.4–50%) after whole pelvic irradiation of 45–50.4 Gy for pelvic malignant disease reported previously in Korean patients with asymptomatic hemorrhoids [11, 12]. Interestingly, few studies from America and Europe have addressed this topic, mainly because both hemorrhoids and AAT are less common in patients in these regions, and the symptoms are usually mild and resolve spontaneously [5, 17, 18]. However, we observed three patients with symptomatic hemorrhoids at the beginning of radiotherapy show severe bleeding that necessitated suspension of radiotherapy for more than 2 weeks to allow medical intervention. On the contrary, in the 11 patients who had a history of surgical removal of hemorrhoids before, no one reported ≥Grade 2 AAT symptoms after radiotherapy. The presence of symptomatic hemorrhoids at the beginning of radiotherapy was expected to be associated with a higher risk for symptom aggravation. Nevertheless, the findings still did not provide a clear idea about preventing AAT. Medical treatment to control anal symptoms in patients with symptomatic hemorrhoids before the initiation of radiotherapy might be one of the possible solutions to reduce the risk and the severity of AAT. However, since the symptoms of AAT are quite different from those of acute radiation-induced proctitis, which are commonly diarrhea and defecation urgency [19–21], it is necessary to distinguish the anal canal from the rectum to seek proper dosimetric recommendations in clinical practice.

Although radiation doses of 45 to 55 Gy in fractions of 1.8 to 2 Gy are considered to be safe for the anal canal [22], patients with hemorrhoids may be troubled even by low radiation doses. The median induction dose for AAT is about 37.5 Gy (38.3 Gy calculated as EQD2) in our study, which is similar to the results of the two studies from Korea (34.1–36.9 Gy) [11, 12]. However, none of the DVH parameters were significant predictors of AAT in their studies, and only V10 or V30 and V40 showed marginal correlations with AAT [11, 12]. Indeed, it is difficult to draw reliable conclusions from two studies with such small sample sizes of 31 and 33 patients, and DVH predictors of AAT should be re-evaluated in larger patient cohorts. In the present study, we enrolled 248 PCa patients treated with pelvic radiotherapy as a training cohort, and our results showed that the maximum and mean dose as well as the V10, V20, V30, V40, V50, V60, and V70 for the anal canal were significantly higher in the AAT group. However, when all these parameters including hemorrhoids were assessed in the multivariate analysis, V20 was the only independent dosimetric factor predicting AAT. Similar results were also observed in our validation cohort.

As noted in our study, the AAT probability increased by only 2.1% for each incremental 1% increase in the anal canal V20, and hemorrhoids remained the most dominant risk factor for AAT. Furthermore, we generated models including hemorrhoids and V20 to predict AAT, and found that any V20 without hemorrhoids had a low +PV, whereas high V20 (> 74.93%) with hemorrhoids had a much higher +PV than low V20 (< 74.93%) with hemorrhoids in both training and validation cohorts. These data indicated that anal canal V20 < 74.93% may serve as a proper dosimetric recommendation to reduce the risk of AAT in PCa patients with hemorrhoids.

Our study has some limitations. First, detailed information regarding the types and grades of hemorrhoids before radiotherapy were not available for most patients; second, the associations between dosimetric parameters and different grades of AAT were not analyzed because of the low incidence of Grade ≥ 2 AAT; third, all the patients of this study performed prostate radiotherapy in prone position, while patients’ position may also contribute to the higher anal dose and the present of AAT. Further studies are still needed to confirm our results.

## Conclusions

AAT is common among PCa patients with hemorrhoids during and after pelvic radiotherapy. Hemorrhoids and anal canal V20 are independent predictors of AAT, which should be carefully considered during treatment planning to minimize the incidence of AAT.

## Data Availability

The dataset used and analysed during the current study are available from the corresponding author on reasonable request.

## Abbreviations

+PV: Positive predictive value
AAT: Acute anal toxicity
DVHs: Dose–volume histograms
IMRT: Intensity-modulated radiotherapy
NAAT: No-acute anal toxicity
PCa: Prostate cancer
PTVs: Planning target volumes
-PV: Negative predictive value
ROC: Receiver operating characteristic
RT: Radiotherapy
V20: Volume received more than 20 Gy
VMAT: Volumetric-modulated arc therapy
